# Restored mutant receptor:Corticoid binding in chaperone complexes by trimethylamine N-oxide

**DOI:** 10.1371/journal.pone.0174183

**Published:** 2017-03-16

**Authors:** Aaron L. Miller, W. Austin Elam, Betty H. Johnson, Shagufta H. Khan, Raj Kumar, E. Brad Thompson

**Affiliations:** 1 Department of Biochemistry and Molecular Biology, Sealy Center for Structural Biology, University of Texas Medical Branch, Galveston, Texas, United States of America; 2 Department of Basic Sciences, Geisinger Commonwealth School of Medicine, Scranton, Pennsylvania, United States of America; Universite de Geneve, SWITZERLAND

## Abstract

Without a glucocorticoid (GC) ligand, the transcription factor glucocorticoid receptor (GR) is largely cytoplasmic, with its GC-binding domain held in high affinity conformation by a cluster of chaperones. Binding a GC causes serial dis- and re-associations with chaperones, translocation of the GR to the nucleus, where it binds to DNA sites and associates with coregulatory proteins and basic transcription complexes. Herein, we describe the effects of a potent protective osmolyte, trimethylamine N-oxide (TMAO), on a conditions-dependent “activation-labile” mutant GR (GR^act/l^), which under GR-activating conditions cannot bind GCs in cells or in cell cytosols. In both cells and cytosols, TMAO restores binding to GR^act/l^ by stabilizing it in complex with chaperones. Cells bathed in much lower concentrations of TMAO than those required *in vitro* show restoration of GC binding, presumably due to intracellular molecular crowding effects.

## Introduction

The GR is a GC-driven transcription factor located mostly in the cytoplasm, where it is bound with several chaperone proteins which keep it in a configuration favorable for binding GC ligands. Binding a GC causes a series of dynamic interchanges of chaperones, culminating in a move to the nucleus, where GR binds to DNA to regulate transcription at specific genes [1]. Since in leukemic lymphoblasts the GC-GR mechanism drives cell apoptosis, we used this as a selective mechanism, isolating clone CEM 3R43 of GR-resistant cells, which bind and retain the GC dexamethasone (Dex) at 4°C but not at 37° [2]. The hGR in these cells is a L753F mutant in its ligand binding domain (LBD) [3]. Thus both the cells and the mutant protein have been termed *activation-labile (act/l)*. Could function be restored to this temperature-and salt-sensitive mutation by use of stabilizing osmolytes?

Stabilizing organic osmolytes are used by many organisms to protect cellular proteins against potentially destabilizing conditions [4]. Such osmolytes can restore proper folding and function to intrinsically disordered (ID) or misfolded proteins, and physico-chemical studies provide the thermodynamic explanation [4,5]. *In vitro*, the osmolyte TMAO stabilizes the ID transaction domain of the normal GR, restoring its ability to bind known partners and coregulatory proteins [6,7]. Others have demonstrated that even when applied externally to cells, relatively low concentrations of TMAO can restore function to mutant proteins [8]. We hypothesized that the *act/l* phenotype could be corrected by use of TMAO.

We show herein that in cell cytosolic extracts, TMAO restore the ability of the mutant GR^act/l^ to bind Dex in activating conditions. Normal GR-specific GC binding at 37°C could also be demonstrated in cells bathed in 50mM TMAO. When tested for restoration of their wild-type GC-driven phenotype, cells showed only slight effects. The explanation appears to be that TMAO stabilizes heteromeric complexes between GR^act/l^ and several of its chaperones.

## Results

### TMAO effect on GR:Dex binding *in vitro*

**Fig 1:**
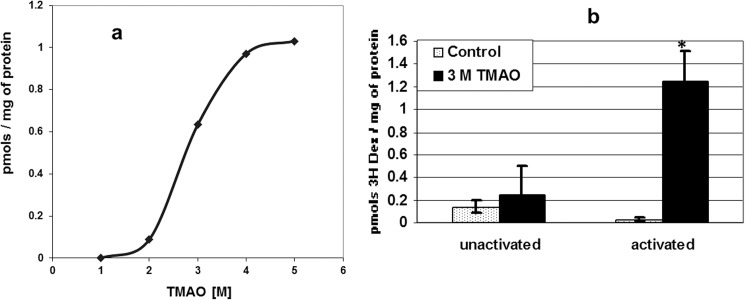
TMAO restores GR-specific binding of a GC to GR^act/l^ *in vitro*. (A) Binding of Dex (50 nM, to avoid non-specific binding) in CEM3R43 cell cytosol as a function of TMAO concentration. (B) Dex binding in the presence or absence of 3M TMAO in CEM 3R43 cell cytosol in GR non-activating (4°) and activating conditions (22°). Activated, Control vs. 3M TMAO significant at p < 0.013 (paired t-test).

CEM-3R43 cells are resistant to Dex-induced apoptosis because they have replaced normal GR with the point mutant GR^act/l^ (L753F). This protein cannot retain the GC Dex, in whole cells at 37°C or under in vitro “activating conditions”, e.g. 22–37°C and physiologic salt concentrations [2]. TMAO has been shown to cause proper folding of various mis- or un-folded proteins, in well-controlled artificial buffers. To see whether TMAO was capable of restoring Dex binding in the complex conditions of a cellular cytosol and activating conditions, we added increasing concentrations of TMAO to crude CEM-3R43 cell lysates, testing for GR-specific ^3^H-Dex binding. Increasing concentrations of TMAO were capable of restoring Dex binding in the complex conditions of a 3R43 cellular cytosol (Fig 1A). The curve comparing restoration of specific Dex binding against TMAO concentration is sigmoidal, with a slope consistent with a multistep cooperative folding effect on the ability of the GR to retain the GC under activating conditions (Fig 1A). In another set of experiments, cell cytosols incubated without TMAO, activating conditions cause loss of Dex binding (Fig 1B, Controls un-activated vs activated). However, when incubated with TMAO, a significant improvement in cytosolic GR-specific binding of Dex is demonstrable (Fig 1B, TMAO, un-activated vs activated). These results show that in principle, TMAO can restore proper binding to the mutant GR^act/l^ in the complex conditions of a cytosol, though the osmolyte concentrations required *in vitro* were high, typical of those required in the dilute solutions employed for more purified protein systems [4,6,7].

### Incubation of CEM 3R43 cells with low levels of TMAO improves Dex binding in cells

**Fig 2:**
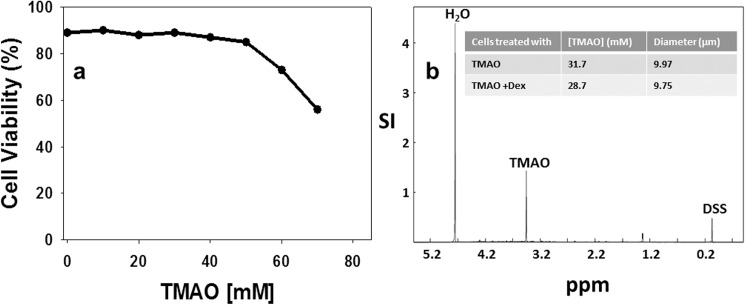
Estimating Concentrations of TMAO in the NMR Sample. (A) Establishing tolerable level of TMAO. Culture medium was brought to Final TMAO concentrations shown and cell viability assayed (trypan blue exclusion). (B) ^1^H-NMR analysis to determine intracellular TMAO levels in cells bathed in medium containing 50mM TMAO. Using a tool in the VNMRJ software, integrals of TMAO peaks were taken in samples and compared to injected DSS reference peak. Molar concentration of TMAO in sample calculated by comparing these two ratios (one of known concentration).

**Fig 3:**
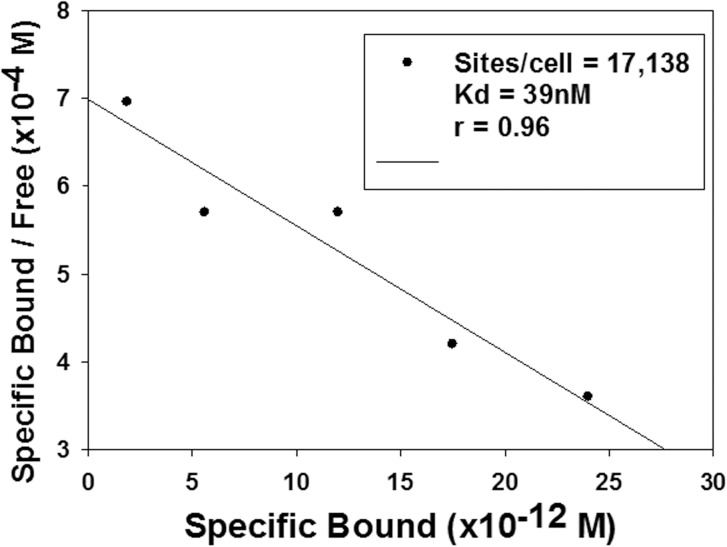
TMAO restores GR-specific binding of a GC to GR^act/l^ in cells. Multi-concentration Dex binding data to specific GR sites in TMAO-bathed cells, plotted by the method of Scatchard. Cellular TMAO concentration was calculated in two ways- based on average protein per cell or average cell volume.

To see whether lower levels of TMAO could have an effect on GR^act/l^ in whole cells, we first determined that the cells could tolerate up to 50 mM TMAO in their culture medium (Fig 2A). We confirmed by use of ^1^D NMR that cellular TMAO levels were similar to those in the medium, i.e. the cells neither concentrated nor excluded the osmolyte (Fig 2B, Table 1). GR^act/l^ levels in 3R43 cells are low, making it difficult to demonstrate GR binding sites; to provide sufficient GR to measure the TMAO effect clearly, 3R43 cells were electroporated with a vector expressing GR^act/l^ and then incubated for 24h at 37°C in medium containing 50mM TMAO. A whole-cell GR-specific binding assay showed that cells in TMAO medium attained about 17,000 Dex binding sites (one site = 1 GR) per cell, K_d_ = 39nM (Fig 3). Without TMAO in this experiment, no evidence for GR binding sites was seen.

**Table 1:** Cellular concentrations of TMAO.

	**Viability**	**Cells/ml**	**Diameter (μm)**	**[TMAO] AP #1**	**[TMAO] AP #2**
Culture #1	98.1%	1.20 x 10^6^	10.20	78.8 mM	78.6 mM
Culture #2	89.4%	0.87 x 10^6^	9.97	31.7 mM	32.6 mM
Culture #2+Dex	87.7%	1.30 x 10^6^	9.75	28.7 mM	25.4 mM
Cellular TMAO uptake based on cell protein content					
	**[TMAO] g TMAO/g cell protein**		**[TMAO] Bradford**	**[TMAO] AP #1**	**[TMAO] AP #2**
Culture #1	2.170		78.6 mM	78.8 mM	70.0mM
Culture #2	0.356		32.6 mM	31.7 mM	28.2 mM
Culture #2+DEX	0.383		25.4 mM	28.7 mM	25.5 mM

After determining the concentration of TMAO in a known sample vol. by NMR, its cellular concentration was calculated by two approaches: 1) count cells and use an avg. cell diameter = 10 μm (from ViCell measurements) to calculate cell volume; 2); calculate the total volume of cell water and divide by amount of TMAO. AP = approach.

### Biologic responses to GCs are only partially restored in TMAO treated cells

**Fig 4:**
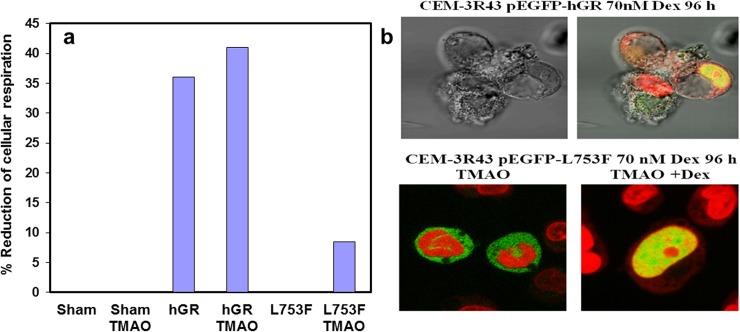
Effects of TMAO on GC-driven GR nuclear translocation and loss of cell viability. (A) Loss of cell viability, estimated by water-soluble tetrazolium mitochondrial respiration assay. To maximize effect, cells were treated with 10^-6^M Dex ±TMAO. “Sham” = electroporation without plasmid; “GR” = wild-type hGR; “L753F” = GR^act/l^. In this experiment, p < 0.05 (paired t-test) only for TMAO vs TMAO + Dex in GR^act/l^ (L753F) transfected cells (n = 3). (B) Confocal microscopy showing transfected EGFP-GR in nuclei of 3R43 cells after 70nM (to enhance assay sensitivity) Dex treatment. Upper Panel left, clump of cells without, and right, same cells with fluorescence; Lower Panel, cells transfected with EGFP GR^*act/l*^. Left, 2 cells in TMAO-supplemented medium, showing cytosolic GR. Right, in TMAO medium plus Dex, a cell showing nuclear GR. Green fluorescence, EGFP fusion GR; red fluorescence, nuclei stained with Draq5.

Cells were transfected by electroporation with pEGFP-hGRα (wild-type) or pEGFP-GR^act/l^ (hGR L753F), grown in medium supplemented with 50mM TMAO, and treated with Dex. Across multiple experiments, GR levels in the TMAO-treated cells ranged from about 2 to 14x the level found in untransfected, wild-type CEM C7-14 cells. We ascribe the range to our use of two expression plasmids with differing promoter strengths and to the variable cellular effects of electroporation, which of itself transfects with high efficiency. Occasionally, at the high GR^act/l^ levels, Dex treatment of the control cells produced a low level of cell death (not in the experiment shown), consistent with a “leaky” GR mutation; addition of TMAO inconsistently enhanced a modest, statistically significant apoptotic effect as assessed by WST-1 assay. An example of this inconsistent result is shown in Fig 4A. As is well known, in normal cells, the bulk of the GR lies in the cytoplasm and is translocated to the nucleus upon addition of Dex [9,10]. Confocal microscopy could demonstrate occasional Dex+TMAO-dependent nuclear localization of GR^*act/l*^. For example, in Fig 4B upper panel are a clump of cells transfected with wt-GR, shown in visible light next to the same cells with fluorescence. In cells transfected with GR^*act/l*^ and treated with TMAO, the bulk of the GR lies in the cytoplasm as it does in cells with wt-GR, but on the addition of Dex, can be found in cell nuclei (Fig 4B; Lower Panel).

**Fig 5:**
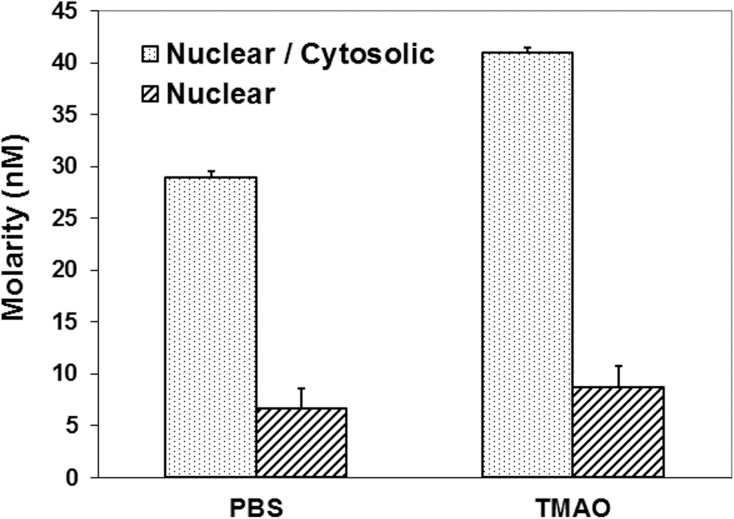
TMAO increases total Dex-GR binding but not % in nuclei. 3R43 cells were transfected with pCMV2 GR(L753F) followed by 50nM H^3^ Dex (subsaturating concentration, to limit non-specific binding). After their separation, radioactivity in nuclear and cytosolic fractions was determined and molarities calculated from the radioactivity data. The combined nuclear + cytosolic GR was higher in the TMAO-incubated cells (p <0.05, paired t-test, n = 3).

Such fluorescence data is difficult to quantify accurately; therefore a cell fractionation assay was carried out. 3R43 cells were transfected with a GR^*act/l*^ expression plasmid, incubated in 50mM-enhanced medium, incubated with 50nM ^3^H Dex (low concentration to limit binding essentially to the GR), collected and separated into nuclear and cytosolic fractions. Radioactivity was measured, and from that, GR content of each fraction was calculated. There was no significant difference observed in the nuclear fractions whereas combined nuclear plus cytosolic fractions showed a significantly higher binding in cells treated with 50 mM TMAO compared with PBS treated cells (Fig 5). Thus, TMAO increases total bound DEX:GR without quantitatively increasing GR movement to the nucleus.

We also tested the effect of TMAO on Dex and GR-driven induction of a model gene. 3R43 cells stably transfected with an MMTV-luciferase construct were bathed in medium supplemented with 50mM TMAO and treated with 10^-6^M Dex or only ethanol vehicle. As is well known, in cells containing wt GR this Dex treatment induces the classic model of MMTV-driven gene expression many fold. Table 2 summarizes the results of 5 experiments assaying the effect of TMAO on induction of luciferase. As the results show, experiment to experiment, addition of TMAO to the medium had only slight and variable effects on the weak (about 2-fold) induction. The number of independent samples varied experiment to experiment, but in experiment 5, where n = 4, with TMAO there clearly was no significant improvement in the induction (p >> 0.05).

**Table 2:** MMTV-luciferase promoter-reporter activity.

Experiment	Replicates	Average Fold Induction No TMAO	Average Fold Induction 50 mM TMAO
1	1/each variable	1.2	1.8
2	2/each variable	2.0	1.6
3	2/each variable	2.5	2.1
4	4 No TMAO, 1 TMAO	1.7	2.3
5	4/each variable	1.7	1.8
**Overall Average Fold Induction**		**1.8**	**1.9**

Results from 5 independent experiments testing induction by 10^-6^M Dex from a stable MMTV-luciferase promoter-reporter construct in 3R43 cells incubated in medium with or without 50mM TMAO. Luciferase values were recorded as RLU/μg cell protein, and fold induction was calculated as RLU (Dex÷control). Replicates = number of independent samples within each experiment. Though numbers of variables differed between experiments, the lack of significant, consistent increase in induction due to TMAO can easily be seen. In experiments 1, 2, 3 and 5 n = 1, 2, 2, and 4, respectively. Experiment 4 had 4 control samples but only one TMAO sample.

### TMAO stabilizes a GR^act/l^:HSP90 complex

**Fig 6:**
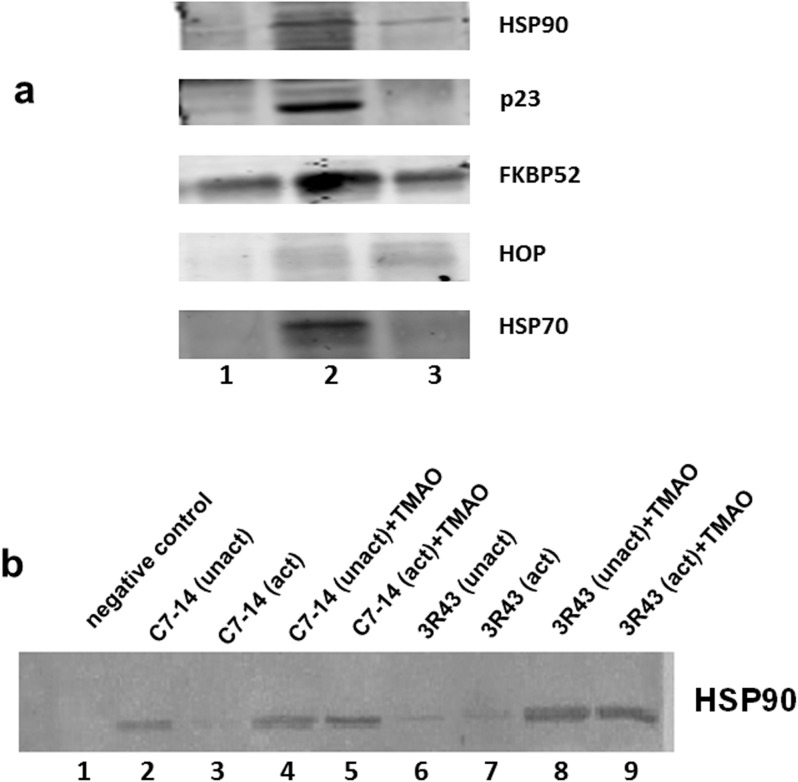
TMAO appears to stabilize GR^act/l^:chaperone complexes. (A) Co-immunoprecipitation (primary antibody to GR) showing the coprecipitation of various known GR chaperones (antibodies specific to the chaperones indicated on right) in 3R43 cell cytosolic extracts. Data from a single blot, equal amounts of protein applied per lane. Lane 1: control extracts (without TMAO); lane 2: cytosolic extracts plus 3M TMAO; and Lane 3: cytosolic extracts from cells treated with 50 mM TMAO and maintained in TMAO throughout. (B) TMAO stabilizes GR:HSP90 complexes in CEM-3R43 and CEM-C7-14 cellular cytosols under both nonactivating (0−4^0^) activating (22^0^) conditions. An immunoadsorption assay was conducted using an anti-GR serum to precipitate the GR:HSP90 complex and blotted against HSP90 antibody. unact = receptor non-activating conditions, act = receptor activating conditions.

One possible reason for the paradox of TMAO-dependent normalization of GR^act/l^:Dex binding, yet lack of GR nuclear translocation and only inconsistent partial apoptosis, is an effect on GR:chaperone interactions. We carried out co-immunoprecipitations using antibodies raised against the GR and several GR chaperones (HSP90, p23, FKBP52, HOP, and HSP70). Cytosolic extracts from CEM-3R43 cells were prepared: 1) in buffer alone; 2) with 3M TMAO; or 3) from cells grown with 50 mM TMAO. Immunoblots suggest that in TMAO-bathed cells and extracts, several GR^act/l^:chaperone interactions may be stabilized (Fig 6A). The HSP:GR interaction was compared between 3R43 and wild-type C7-14 cell extracts. As expected, in C7-14 cells, the GR appears to form a strong complex with HSP90 under non-activating conditions (Fig 6B; lane 2), whereas under activating conditions (Fig 6B; lane 3), the HSP90 in the complex is mostly dissociated. Under similar conditions in 3R43 cells, there is only a weak interaction between the GR and HSP90 in both conditions (Fig 6B; lanes 6 and 7), suggesting that in these cells the act/l GR is unable to form a strong complex with HSP90. In the presence of 3M TMAO, under both conditions in 3R43 cell extracts GR^*act/l*^ interaction with HSP90 is increased considerably (Fig 6B; lanes 8,9). Interestingly, GR:HSP90 and GR(L753F):HSP90 complexes are still intact in this high TMAO concentration even in activation conditions (Fig 6B; lanes 5 and 9) in both C7-14 and 3R43 cells.

## Discussion

TMAO and other “protective” organic osmolytes exist in a wide variety of eukaryotic systems, where they act to prevent denaturation of cellular proteins from extremes of temperature, dehydration, osmotic stresses and other potentially harmful conditions. The genes responsible for production of these compounds are under strict control [11]. Protective osmolytes act through the balance of aquaphobic and aquaphilic effects on protein backbone and amino acid side chains and can restore misfolded or unfolded proteins to their normal, functional conformations [4]. When applied to steroid hormone receptors, protective osmolytes cause folding of their ID N-terminal domains [6,12,13]. SHRs must bind certain other proteins to function, and such binding is restored or greatly enhanced in SHR N-terminal domains treated with protective osmolytes.

In the absence of GCs, the C-terminal LBDs of SHRs are not well folded; intracellularly, when steroid ligand is not present, the SHR LBD is held in its proper ligand-binding, 12-helical configuration by a group of chaperones, including HSP90 and 70, FKBP 51 and 52, HOP and p23. These and additional chaperones bind to the LBD and each other, with HSPs 90 and 70 as critical platforms for formation of the heteromeric complexes [14]. Upon steroid ligand entering its LBD pocket in the GR, the chaperones are shed or exchanged, and the largely cytoplasmic GR is able to enter the nucleus, possibly still in concert with certain chaperones [15]. There, it is able to bind DNA and various cofactors, so as to regulate gene expression.

**Fig 7:**
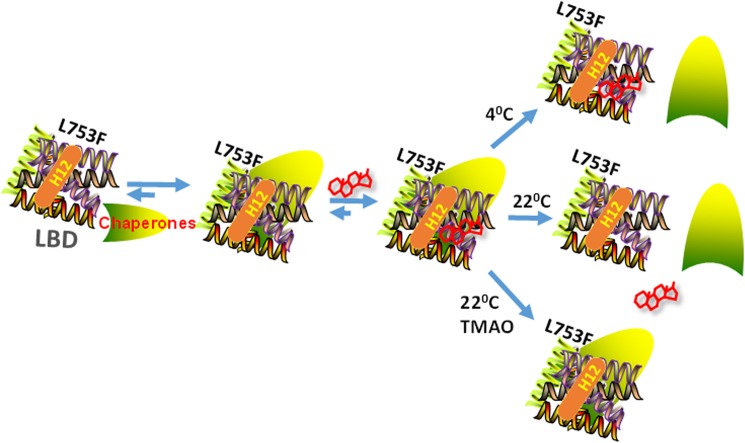
A model for TMAO cellular effects in 3R43 cells. The GR^act/l^ L753F LBD is shown as a bundle of helices. Adding chaperones (green symbol) allows a configuration favorable for high-affinity steroid binding. At 4^0^ (in low-salt buffer) the mutant GR retains steroid; at 22^0^ (up to 37^0^) plus 0.2 M up to isotonic salt, steroid and chaperones fail to bind; TMAO preserves the chaperone interaction and thus steroid binding.

Since the *act/l* mutation, L753F, is in the LBD, it seemed likely that the failure of GR^act/l^ to properly retain the ligand Dex was due to a failure of the chaperone:GR system. We postulated that TMAO, one of the most powerful protective osmolytes, would cause the stabilized GR LBD- even when released from its chaperones, to retain a conformation capable of continuing to bind steroid and hence, to carry out its functions of gene regulation leading to apoptosis. Prior reports showed significant restoration of function to membrane salt channels in cystic fibrosis cells bathed in modest concentrations of TMAO. Our results, however, only partially supported our hypothesis. TMAO did stabilize steroid:GR binding, but only partial GR functions of apoptosis or gene induction. The reason appears to be that a GR^act/l^:chaperone complex favorable for steroid binding was stabilized. TMAO acts both on the GR and on HSP90 [16]; so both effects may be relevant. The stabilized GR:chaperone complex presumably prevented the complex dissociations and re-associations necessary for full GR actions (Fig 7).

TMAO interacts with both peptide backbone and amino acid side chains. The net physico-chemical effect on protein surfaces exposed to and driven away from water determines whether the protein achieves proper structure [4]. In the case of GR^act/l^, addition of TMAO may thermodynamically stabilize the GR:chaperone complex and destabilize unbound/disordered GR states, driving the GR to populate the complex within cells.

As to why a low level of intracellular TMAO achieves GR:HSP90 stabilization, we note that low TMAO levels have been effective in other *in vitro* and *in vivo* systems [17]. The likely reason is the normal intracellular molecular crowding [18–20]. Macromolecules account for about 40% of cell volume; thus all intracellular reagents are greatly concentrated, with both favorable and unfavorable effects on protein folding [21–23]. The pro-folding effects of organic osmolytes may be intensified in the already molecularly crowded conditions of a cell [24]. With respect to signal transduction pathways, molecular crowding is potentially of great significance [25]. We speculate that even in ~50 mM concentrations of TMAO, pre-existing intracellular crowding promotes stabilization of the GR^*act/l*^:chaperone complex, preserving GR in a configuration favorable for high affinity GC binding but preventing full physiological functions.

Our results give proof of principle that an osmolyte applied extracellularly can correct the ligand binding defect in a mutant GR, probably due to stabilization of the GR:chaperone complex, which functions to keep the GR LBD in a configuration favorable for binding steroid. We show that when TMAO is taken within cells, the intra- and extracellular concentrations are essentially isosmotic. The mechanism that corrected the binding defect, however, prevented correction of the cell phenotype. Despite the fact that external TMAO has been applied to correct membrane protein function in cystic fibrosis, the actions of TMAO on multiple proteins may disrupt intracellular signaling networks [17]. However, since various osmolytes- polyols, amino acids, sugars, and methylamines can have differing, even synergistic, effects on proteins, the data encourage further exploration for basic concepts and possible practical uses.

## Materials and methods

### Materials

Unlabeled Dex and all other chemicals were purchased from Sigma-Aldrich (St. Louis, MO). ^3^H Dex was from Amersham Biosciences (Piscatoway, NJ, 40 Ci/mmol). Trimethylamine N-oxide, (CH_3_)_3_ NO, was purified when necessary, as described [26]. Water soluble tetrazolium (WST) was obtained in a kit (Roche/Boehringer-Mannheim, Indianapolis, IN).

### Use of specific differing Dex concentrations

Three concentrations of Dex were employed for differing experiments and purposes. Because of the high affinity of Dex for GR as opposed to low-affinity, non-specific binding sites in the cell, 50nM Dex binds essentially only to GR. At 70nM, primary responses to Dex in cells are just entering the steep part of the dose-response curve. We therefore used this concentration in expectation of increasing assay sensitivity. At 10^-6^M Dex, the GR binding site is fully filled; at this steroid concentration, an effect of TMAO might best be seen if only a fraction of GR^*act/*^ is converted to a form proper for GC binding.

### Cell culture

The Dex-sensitive clone CEM C7 was cultured from the human lymphobast line CEM CCRF (2); CEM-C7-14 and CEM-3R43 are sub-clones of CEM C7. For some experiments, CEM-3R43 cells stably transfected with the promoter-reporter construct MMTV-Luc were used. This construct contains a classic GC/GR-responsive promoter driving a luciferase reporter gene. All cells were grown in RPMI 1640 (Cellgro, from Mediatech Herndon, VA) supplemented with 5% fetal bovine serum (Atlanta Biologicals, Norcross GA) and divided every 3–4 days to maintain logarithmic growth. Cells were grown in a Forma tissue culture incubator (Thermo Electron Corporation, Franklin, MA) in a humidified atmosphere of 95% air / 5% CO_2_ at 37°C.

### Cytosolic GR:GC binding

Mid-logarithmically growing CEM-3R43 cells were pelleted at 1,000 rpm for 15 minutes at 22°C. Cells were washed once with phosphate buffered saline, pH 7.4 (PBS, Invitrogen, Carlsbad, CA) at room temperature, transferred to clear micro-tubes and re-pelleted. An equal volume of 4°C lysis buffer (0.01 M Tris, 0.0015 mM EDTA, 0.5 mM DTT, 1x protease inhibitor cocktail, pH 7.4) ± 3.0 M TMAO was added, the micro-tube contents gently mixed, and the tubes placed on ice. The lysates were then twice frozen at –80°C and thawed on ice. Cytosols were prepared by centrifuging the samples in a table-top ultracentrifuge (Beckman Coulter, Model TL-100, Miami, FL) at 45,000 rpm for 30 minutes at 2°C. Clarified cytosol was then carefully transferred to fresh cold micro-tubes. For Dex-binding in non-activating conditions, triplicate cytosols ±TMAO were incubated in the presence of 50 nM ^3^H Dex (uncompeted samples) and 50 nM ^3^H Dex plus a 100-fold excess of cold Dex (competed samples) for 24 h. For binding in activating conditions, cytosols were incubated at 22°C with 0.2 M KCl for 30 minutes prior to being placed at 4°C for 24 h. A 100 μl of hydroxyapatite slurry (hydroxyapatite hydrated with lysis buffer ± 3.0 M TMAO) was added to each sample. Samples were then placed at 4°C for 30 minutes, pelleted at 1,000 rpm for 1 minute at 4°C in a table-top micro-centrifuge (Beckman Coulter, Model Microfuge R). Samples were washed 3 times with the above buffer without hydroxyapatite and centrifuged. The hydroxyapatite pellet was then placed in 10 mL of scintillation cocktail and its radioativity quantitated by scintillation counting (Beckman Coulter, Model LS 5801). The specifically bound GC was expressed as pmol ^3^H Dex/mg cytosolic protein.

### Whole cell GR:GC binding

Cells were transfected as described below and cultured in medium containing 50 mM TMAO or an equivalent volume of PBS. The cells were pelleted and re-suspended in 37°C RPMI 1640 medium plus 5% FBS plus 25 mM Tricine with PBS vehicle or 50 mM TMAO. 1.0 mL aliquots of cell suspensions were transferred to borosilicate glass test tubes (12x75 mm) containing ^3^H Dex serially diluted from 100 nM to 1.5 nM ± 200-fold molar excess cold Dex. 50 nM concentration was used to avoid non-specific binding. The samples were incubated and washed as in the cytosolic binding assays. After the final wash the samples were resuspended in 1.6 mL of HBSS. Cells in 200 μl of each sample were counted by Vi-cell. One ml of sample was added to 10 mL of scintillation cocktail and its radioactivity quantitated. Sites/cell and K_d_ were calculated using the method of Scatchard [27].

### Nuclear/Cytosolic distribution of GR-bound GC

Logarithmically growing CEM-3R43 cells were collected by centrifugation, washed with 10 mL of sterile 37°C PBS and recollected. Cells were resuspended in serum-free RPMI 1640 to a density of 1x10^7^ viable cells/mL. Aliquots (800 μl) of the suspension were placed into 0.4 cm gap electroporation cuvettes (Bio-Rad, Hercules, CA) containing 15 μg of phCMV_2_-GR(L753F) vector (Gene Therapy Systems, San Diego, CA) prepared using a Qiagen maxi-prep kit (Qiagen, Valencia, CA). Cuvettes were electroporated using 500 μF and 300 V in a Gene Pulser II (Bio-Rad). Electroporated cells were diluted in 10 mL (per cuvette) of RPMI 1640 supplemented with 5% FBS and re-cultured. Three hours after transfection, 50 ng/ml phorbol myristate acetate and 1 μg/mL phytohemagglutin were added to increase transgene expression. Cells were then divided into two groups and received either PBS or 50 mM TMAO. 24 h after transfection, cells were counted using an automated Trypan blue exclusion system (Vi-cell, Beckman Coulter) and adjusted to a concentration of 6x10^5^ cells/mL using 37°C RPMI 1640, 5% FBS, 25 mM Tricine ± 50 mM TMAO in a total volume of 2 mL, which was divided equally between two 12 x 75 mm borosilicate glass test tubes. One tube “un-competed” received 70 nM ^3^H Dex and the other, “competed” received 70 nM ^3^H Dex plus a 200-fold molar excess of unlabeled Dex. Cells were placed in a tissue culture incubator at 37°C for 1 h. Three mL of Hanks balanced salt solution (HBSS) (Cellgro) ± 50 mM TMAO were then added, and the samples were centrifuged at 1,200 rpm for 1 minute at room temperature in a Beckman table-top centrifuge. After three additional washes the cells were transferred to micro-tubes and centrifuged. The moist cell pellets were flash frozen in a dry ice/methanol bath and placed at –80°C for 10 minutes. After thawing 4°C for 15 minutes, all solutions and samples were kept at 4°C for the remainder of the experiment. 1.1 mL of 1.5 mM MgCl_2_ plus ± 50 mM TMAO was added with vigorous for 30 seconds. One-half mL of the sample (nuclear + cytosolic) was added to 10 mL of scintillation cocktail (Fisher Scientific, Austin, TX) and evaluated using a Beckman liquid scintillation counter. Nuclei were separated by centrifuging the samples on a Beckman table-top microfuge at 9,000xg for 4 minutes at 4°C. The supernatant was aspirated and the bottom of the tube containing the crude nuclear pellet was cut off and placed into 10 mL of scintillation cocktail and quantitated as above.

### Nuclear translocation of GR^act/l^ (L753F)

CEM-3R43 cells in mid-logarithmic growth were transfected with pEGFP-GR (L753F) (BD Bioscience Pharmingen, San Diego, CA) by pelleting the suspension at 1,000 rpm for 10 minutes at 25°C in a Beckman table-top centrifuge. Cells were resuspended in 10 mL of 37°C PBS and centrifuged as above for 5 minutes. Cells were adjusted to a concentration of 1.0x10^7^ cells/mL in a serum free RPMI 1640 medium. 800μl aliquots of the cell suspension were electroporated with the GR^act/l^ plasmid, as above. The transfected cells were transferred to 25 cm^2^ tissue culture flasks containing 37°C RPMI 1640 medium plus 5% FBS and placed in the incubator. 3h after electroporation, PBS vehicle or 50 mM TMAO, plus 50 ng/mL of phorbol myristate acetate (PMA) and 1 μg/mL of phyto-hemagglutinin (PHA) (Sigma-Aldrich, St. Louis, MO) were added to each flask. 24 h after transfection, cells were counted using automated trypan blue exclusion (Vi-cell, Beckman) and diluted to a concentration of 2.0x10^5^ cells/mL using RPMI 1640 plus 5% FBS containing PBS vehicle or 50 mM TMAO at 37°C. Ethanol vehicle or 70 nM Dex was added to the appropriate treatments. 24 h after the addition of Dex, cells were pelleted as above for 10 minutes, transferred to micro-tubes, resuspended in 4% paraformaldehyde/PBS and fixed for 5 minutes. Fixed cells were washed 3 times with PBS and resuspended to a final volume of 100 μl PBS. Nuclei were stained by adding Draq 5 (Axxora San Diego, CA) to a final concentration of 10 μM. Cells were diluted to a volume of 1mL in PBS, placed on a 0.17 mm thick 25 mm circle cover slip (Fisher Scientific) and analyzed by a LSM 510 Meta laser-scanning confocal microscope (Zeiss, Thornwood, NY) using a plan-apocromat 63/1.4 oil objective with both a band path filter of 505-530nm and a long pass filter 650nm for EGFP and Draq 5 imaging, respectively. 10 random fields for each treatment were chosen. Metamorph (Universal Imaging Corporation, Downington, PA) imaging software was used to draw a boundary around the Draq 5 stained nuclei. EGFP fluorescence was then quantitated within this boundary. The data obtained were transferred into an Excel (Microsoft Corporation) spreadsheet, and after subtracting background was subtracted, the results were displayed as histograms, (Sigma Plot, Rockware Inc, Golden, CO).

### Evaluation of apoptosis

CEM-3R43 cells were transiently transfected with pEGFP-hGRα or pEGFP-L753F and subsequently incubated for 24 hours in medium supplemented with 50 mM TMAO or an equivalent amount of PBS vehicle. Cell viability was estimated by WST-1 reagent following the instructions of the manufacturer (Roche Diagnostics/Boehringer Mannheim, Indianapolis, IN). After adding Dex or vehicle, the cultures were incubated for 96h; then 200 μl aliquots from each treatment were transferred to a 96 well plate. 20 μl of freshly prepared and filtered WST-1 reagent was added, mixed with the suspension, and the cells were incubated overnight at 37°C in the dark. The absorbance of the stable formazan product was measured at 450 nm, with a background reference wavelength of 595 nm.

### Immunoadsorption assay

From cytosolic extracts of CEM-3R43 or CEM-C7-14 cells, 60μl were prepared buffer (0.01 M Tris, 0.0015 mM EDTA, 0.5 mM DTT, 1x protease inhibitor cocktail, pH 7.4) with or without 3 M TMAO. In another set, cytosolic extracts were prepared from cells treated with 50 mM TMAO as described in whole cell GR:GC binding assay (above). Cytosols were divided equally and incubated under “non-activating” and “activating” conditions, while maintaining the TMAO concentration. The preparations then had 5 μl of GR antibody (Affinity Bioreagents, Golden, CO) added, followed by incubation for an additional 1 hour at 4°C. Each sample was raised to a final volume of 0.5 ml, and 25 μl of protein agarose conjugate were added, followed by incubation for a further 2 hours. Pellets were collected by centrifugation, and washed thoroughly. The beads were resuspended in SDS-sample buffer and boiled for 5 min. After SDS-PAGE gel electrophoresis, the proteins were transferred onto a PVDF membrane (Bio-Rad) using a semi-dry electroblotter (Bio-Rad) and immunoblotted using an antibodies raised against HSP90 (AC88) (Santa Cruz Biotechnology, Santa Cruz, CA), or the other chaperones indicated (kind gift of Dr. David Toft).

### Determination of TMAO concentration in intact cells

CEM-3R43 cells were cultured in RPMI 1640 supplemented with 5% fetal bovine serum. At the point of mid-log growth TMAO was added to the medium at the final concentration of 50 mM. Following 24 hour incubation, cells were collected, washed twice with PBS, lysed with 2.5% Percloric Acid (PCA) and sonicated in ice three times for 10 seconds. After centrifugation, supernatants were collected and dried overnight, resuspended in 500 μL of water containing 20% D_2_O. The sample was then applied to ^1^H-NMR analysis on a Varian 400 MHz spectroscope, and data collected. At the end of the run, using a tool in the VNMRJ software, integrals of TMAO peaks were taken in samples and compared to injected 100μM DSS reference peak. Cellular concentrations of TMAO were calculated in three ways, which gave concordant results: 1, by carefully counting cells and relating NMR results to average cell volume or water; 2, by determining protein concentration/cell and then determining protein concentrations in samples.
